# The effects of pelvic ring injuries on quality of life, physical, and mental health: results of a 2-year prospective cohort study

**DOI:** 10.1007/s00068-022-01893-3

**Published:** 2022-03-05

**Authors:** Hester Banierink, Kaj ten Duis, Anne M. L. Meesters, Nymke M. Trouwborst, Erik Heineman, Klaus W. Wendt, Joris J. W. Ploegmakers, Inge H. F. Reininga, Frank F. A. IJpma

**Affiliations:** 1 Department of Trauma Surgery, University Medical Center Groningen, University of Groningen, Groningen, The Netherlands; 2 Department of Surgery, University Medical Center Groningen, University of Groningen, Groningen, The Netherlands; 3 Department of Orthopedics, University Medical Center Groningen, University of Groningen, Groningen, The Netherlands; 4 Emergency Care Network Northern Netherlands (AZNN), Northern Netherlands Trauma Registry, Groningen, The Netherlands

**Keywords:** Pelvic ring injury, Physical functioning, Quality of life, Mental health, SMFA, EQ-5D

## Abstract

**Purpose:**

Pelvic ring injuries are known to affect the patients’ daily life in terms of physical functioning and quality of life (QoL). Still, prospective studies on the patient’s perception over the first 2 years of rehabilitation are lacking. Therefore, patients cannot be properly informed about whether or when they will return to their pre-existing level of physical functioning and QoL.

**Methods:**

A prospective longitudinal cohort study was performed over a 4-year period including all consecutive patients above 18 years who sustained a pelvic ring injury in a level 1 trauma center. Validated patient-reported outcome measures (PROMs) were used to assess physical functioning (SMFA) and QoL (EQ-5D) at baseline (recalled pre-injury score), 6 weeks, 3 months, 6 months, 1 year and 2 years after the injury. It was assessed whether patients had fully recovered by comparing follow-up scores to baseline PROMs. Binary logistic regression analysis was used to identify independent predictors for patients who did not fully recover. Most experienced difficulties at 3 months and 1 year were identified by analyzing the highest reported scores on individual items of the SMFA.

**Results:**

A total of 297 patients with a pelvic ring injury were identified of which 189 were eligible for follow-up and 154 (82%) responded. Median SMFA function score at 3 months, 1 and 2 years was 70, 78 and 88, respectively, compared to 96 out of 100 before the injury. Median SMFA bother score was 67, 79 and 88, respectively. Median EQ-5D score at 3 months, 1 and 2 years was 0.61, 0.81 and 0.85, respectively, compared to 1 (maximum achievable) before the injury. After 1 and 2 years of follow-up, 61% and 75% of the patients fully “recovered” in physical functioning and 52% and 71% fully recovered in terms of QoL. Female gender and high-energy trauma were independent predictors for not fully recovering after 1 year. After 3 months of follow-up, 54% of patients reported severe difficulties with recreational activities, whereas after 1 year, most experienced difficulties (31% of patients) concerned heavy house or yard work. Moreover, after 3 months and 1 year, 44% and 27% of patients reported feeling physically disabled.

**Conclusion:**

Pelvic ring injuries have a large impact on the patients’ daily life in the first 2 years of rehabilitation. Directly after the injury, physical functioning and QoL decrease strongly but then gradually improve over a 2-year period with about 75% of patients fully recovering. Female gender and high-energy trauma are shown to be independent predictors for not fully recovering. After 3 months, patients experience difficulties with both the physical and mental effects of the injury which continue to be present after 1 year.

**Supplementary Information:**

The online version contains supplementary material available at 10.1007/s00068-022-01893-3.

## Introduction

Pelvic ring injuries have an estimated annual incidence of 14–37 per 100,000 inhabitants [1, 2]. In the younger population, high-energy trauma like traffic accidents are often the cause of injury [3], whereas in the fragile elderly, low-energy trauma like a fall from standing is more likely to occur. Pelvic ring injuries can have a serious impact on the patient’s physical functioning and quality of life (QoL) [4], especially during the first months of rehabilitation. They often coincide with a long period of impaired mobilization and pain [5].

Although there has been a shift in terms of outcome assessment with increasing emphasis on patient-reported outcome, prospective follow-up studies on pelvic ring injuries are scarce [6]. A systematic review revealed that some retrospective studies reported that patient-reported physical functioning and QoL seem fair and tend to improve after the injury [7]. However, most studies had several methodological limitations [7]. First, patient numbers were often small and groups were heterogeneous in terms of age, type of injury and treatment. Second, a large number of different generic and pelvic-specific Patient-Reported Outcome Measures (PROMs) were used, while most of these were not validated. At last, most studies used a retrospective design, thus lacking information on the pre-injury health status and rehabilitation period [7]. Still, it is unknown whether or when patients return to their pre-existing level of physical functioning and QoL. As a result, patients cannot be informed properly about prognosis because clinicians lack knowledge about the early recovery of physical functioning and QoL after a pelvic ring injury.

A prospective cohort study was performed concerning the short-term effects of pelvic ring injuries on patient-reported physical functioning and QoL. Based on this information, patients can be informed properly about what to expect from the rehabilitation period in terms of when or whether they will regain their normal life again. Hence, the research questions of this study include: (1) what is the course of recovery in terms of physical functioning and QoL within the first 2 years after a pelvic ring injury?; (2) which patient characteristics are predictive for a decrease in physical functioning and QoL 1 year after the injury?; and (3) from a patient perspective, what are the most experienced difficulties in life at 3 months and 1 year of follow-up after a pelvic ring injury?

## Patients and methods

### Patients

A prospective longitudinal cohort study was performed, including all consecutive adult patients (above 18 years of age) who had been treated for a pelvic ring injury at a level-1 trauma center between January 2017 and June 2021. Data on the patients’ characteristics were prospectively collected and directly entered into the database upon clinical presentation. These include information about the injury, treatment, complications and mortality. Additional data were retrieved from the Dutch Trauma Registry [8], concerning injury severity in terms of the Injury Severity Score (ISS) [9]. Subsequently, two trauma surgeons with ample experience in pelvic ring injury surgery assessed the radiographic images (plain anteroposterior, inlet and outlet radiographs and CT scans) of all the patients and classified the pelvic ring injuries into type A, B and C injuries according to the AO/OTA classification [10]. The local Medical Ethical Review Board reviewed the methods employed and waived further need for approval (METc 2017/543).

### Patient-reported physical functioning and quality of life

All patients who survived the initial injury, without cognitive disorders and who were able to speak and understand the Dutch language were informed about the study and asked to participate. Physical functioning was measured with the Short Musculoskeletal Function Assessment (SMFA). The SMFA questionnaire consists of 46 items which are scored on a 5-item Likert scale. It was designed to assess the functional status of patients with various musculoskeletal disorders and injuries. Two indices (function and bother index) [11] and, additionally, four subscales (upper extremity dysfunction, lower extremity dysfunction, problems with daily activities, and mental and emotional problems) can be calculated [12]. Scores are calculated by summing up the scores on the individual items and transforming scores on a range from zero to 100, with higher scores indicating better function. The SMFA-NL has been shown to be a valid and reliable questionnaire for the assessment of physical functioning in injured patients [12, 13]. Quality of life was assessed with the EuroQol-5D (EQ-5D [14]). The EQ-5D is a brief questionnaire that measures health-related quality of life based on five dimensions of health: mobility, self-care, usual activities, pain/discomfort and anxiety/depression [15]. Items are scored on a 5-item Likert scale through which patients can delineate whether they have (1) no problems, (2) slight problems, (3) moderate problems, (4) severe problems or (5) extreme problems. Based on these values, a utility score ranging from 0 to 1 was formed, with higher scores indicating better function. The EQ-5D has been shown to be a valid and reliable questionnaire in injured patients [16].

The SMFA-NL and EQ-5D were administered at the following time points: During hospital admission (assessment of recalled pre-injury status), 3 months, 6 months, 1 year and 2 years after the injury. Additionally, the EQ-5D was also administered at 6 weeks of follow-up (FU). The PROMs were digitally distributed through a secured system, RoQua, and linked to the electronic patient files. This system provides a personal code which is linked to a secure website and allowed patients to complete the digital PROMs at home or during their follow-up visits at the pelvic outpatient clinic.

### Statistical analysis

Descriptive statistics were performed to present patient and injury characteristics such as injury mechanism, fracture patterns and treatment methods. Means and standard deviations were calculated from the normally distributed data and the median and interquartile range (IQR) from not-normally distributed data. Either Chi-Square test, independent samples *t*-tests or Mann–Whitney *U* tests were performed accordingly to assess differences in characteristics between included patients and patients that were not eligible or declined to participate.

To gain insight into the decrease in physical functioning (SMFA) and QoL (EQ-5D) at every time point of FU relative to their pre-injury status, the scores on the SMFA and EQ-5D were expressed as a percentage of the pre-injury score. Additionally, each patient was classified as “recovered” in terms of physical functioning when his/her score on the SMFA Indices and subscales was 15 points or less below the recalled pre-injury SMFA scores. Similar, for the EQ-5D, patients were classified as “recovered” in terms of quality of life when his/her score on the EQ-5D was 0.15 or less below the re-called pre-injury score. Independent predictors for patients that were classified as not being recovered as measured by the SMFA function and bother index and the EQ-5D after 1 year of follow-up, were analyzed by using a binary logistic regression analysis (backward selection procedure, *p*-out = 0.20). Gender (female/male), age (< 65/ ≥ 65), injury mechanism (LET/HET), ISS (< 16/ ≥ 16), injury type (AO type A/B/C) and complications (yes/no) were evaluated for being possible predictors. The results of the final model are presented as odds ratios (ORs) with their corresponding 95%CI, and P-values. To be able to identify in which domains or activities people felt most limited and whether the (level of) limitations on these domains/activities change over time, the five individual items of the SMFA at which most patients experienced severe problems at 3 months and 1 year of follow-up were reported. Data were analysed using the IBM SPSS software, version 23.0 for Windows (IBM Corporation, Armonk, NY). Statistical significance was set at *P* ≤ 0.05.

## Results

### Study population

**Fig. 1:**
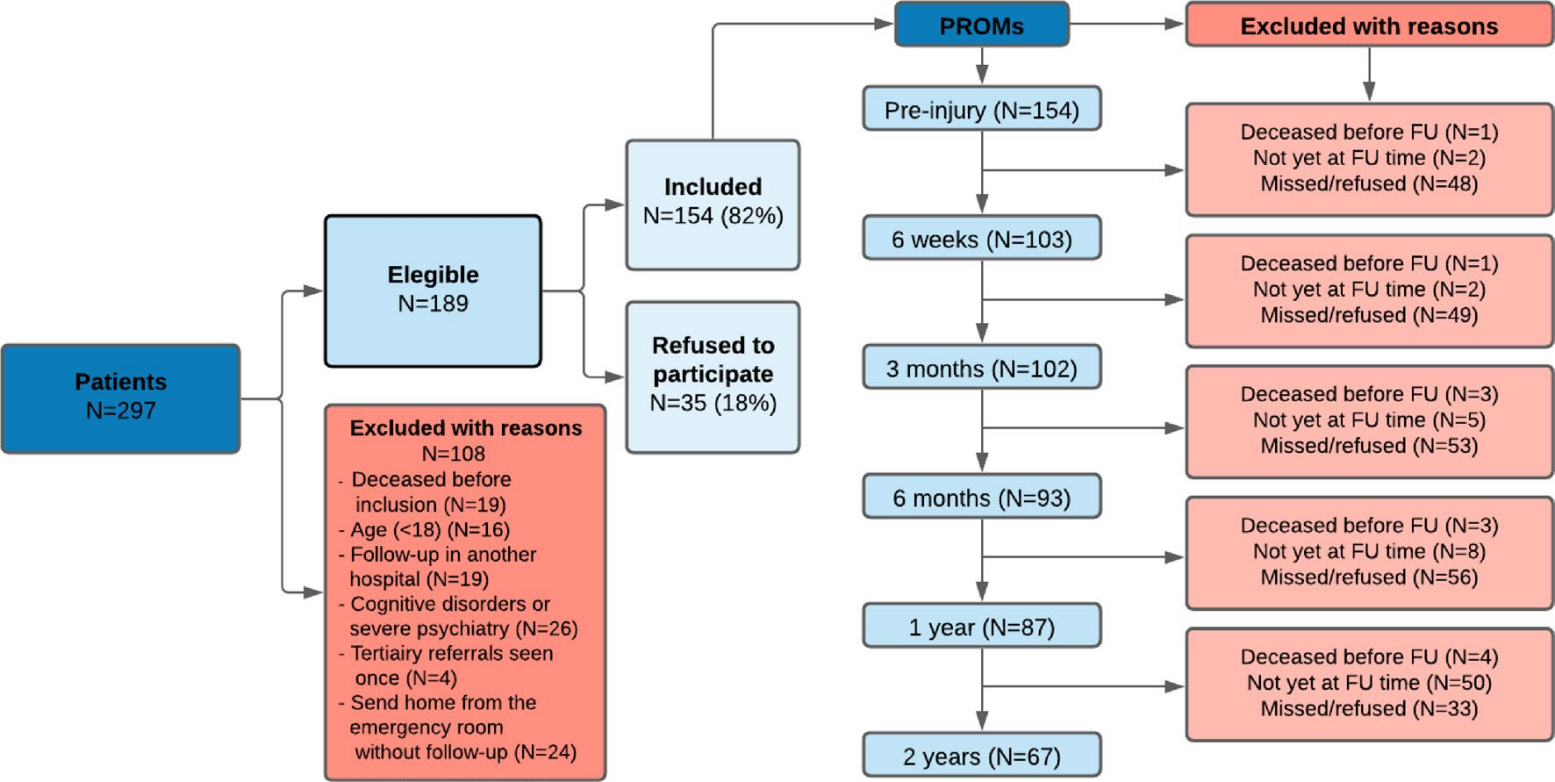
Flowchart of patient inclusion

A total of 297 patients with a pelvic ring injury were treated during the study period of 4 years. One-hundred and eight patients (36%) were excluded due to reasons mentioned in Fig. 1. One-hundred and eighty-nine patients were eligible for follow-up of which 35 refused (18%) to participate. Eventually, 154 patients (82%) filled out one or more follow-up questionnaires. A non-response analysis between the responders and the patients that refused to participate revealed several differences. Patients that were included in the follow-up had a lower median ISS of 13 (IQR 8–20) compared to 17 (IQR 8–37) of patients that were not included (*p* = 0.01). Furthermore, included patients differed from non-included patients in injury types (28% vs. 51% type A, 57% vs. 40% type B and 15% vs. 9% type C); *p* < 0.001, associated lower extremity injuries (13% vs. 27%; *p* = 0.004), operative treatment (28% vs. 8%; *p* < 0.001) and emergency laparotomy (1% vs. 8%; *p* = 0.002). Patient characteristics are shown in Table 1.

**Table 1:** Patient characteristics

	All patients (*N* = 297)
Female, *n* (%)	157 (53)
Age at the time of injury (mean ± SD)	57 ± 22
HET, *n* (%)	115 (39)
Injury Severity Score (ISS) median (IQR)	14 (8–26)
Injury type, *n* (%)	
Type A	117 (39)
Type B	144 (49)
Type C	36 (12)
Isolated pelvic ring injury, *n* (%)	123 (41)
Associated lower extremity injuries, *n* (%)	59 (20)
Operative treatment, *n* (%)	53 (18)
Emergency laparotomy, *n* (%)	12 (4)
External fixator, *n* (%)	14 (5)
Embolization, *n* (%)	7 (2)
Complications < 30 days, *n* (%)	37 (13)
Late onset complications, *n* (%)	8 (3)
Deceased, *n* (%)	53 (18)
< 30 days	19 (6)
< 1 year	38 (13)

### Patient-reported physical functioning and QoL

**Fig. 2:**
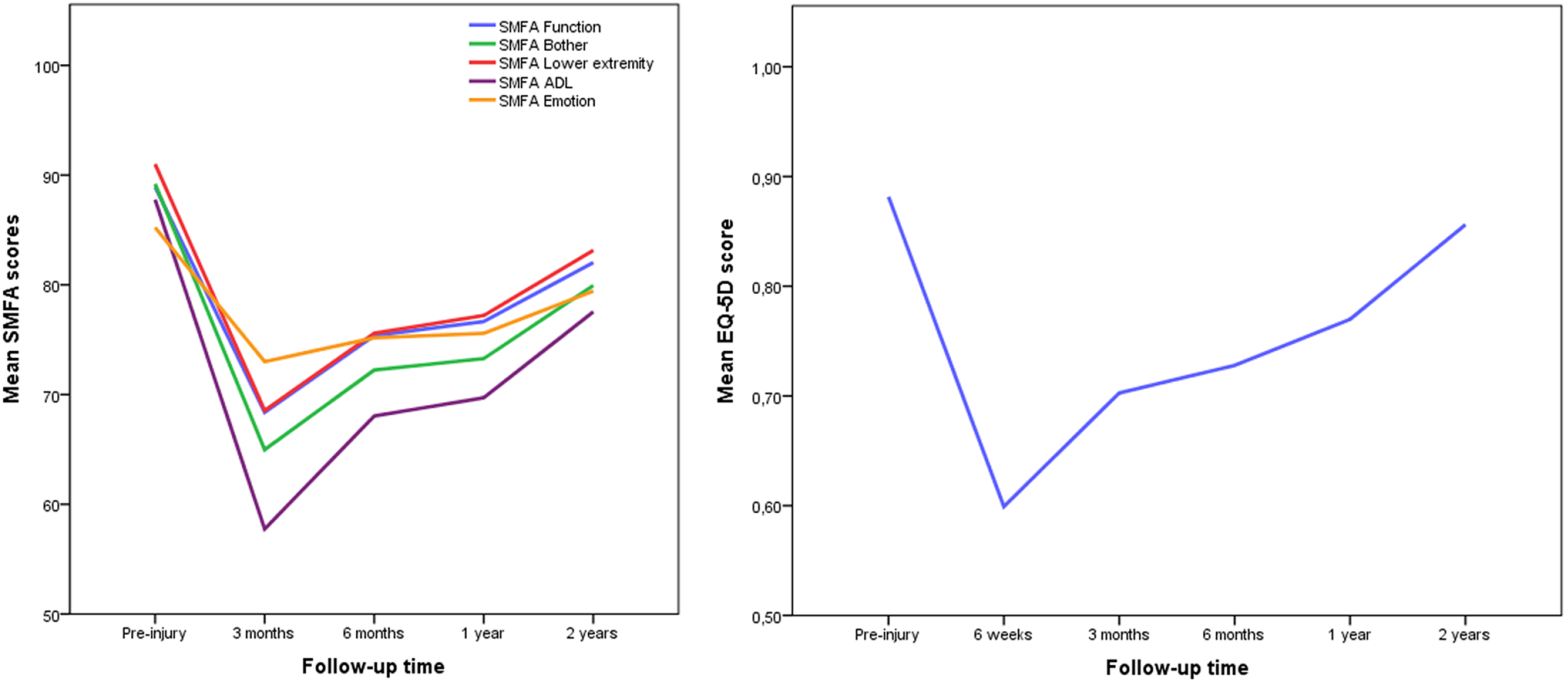
Outcome of PROMs for the SMFA and EQ-5D at different time points

Figure 2 graphically shows the development of physical functioning and QoL during the first 2 years of the rehabilitation phase. All PROMs results are presented in Table 2 together with the median percentage of recovery at every time point of follow-up. For the function index of the SMFA, 61% of patients regained a full recovery at 1 year, and 75% at 2 years of follow-up (Table 2, last column). For the bother index of the SMFA, 57% (1 year) and 68% (2 years) regained full recovery. After 1 year and 2 years of follow-up, respectively 52% and 71% had regained full recovery in QoL as measured by the EQ-5D. Additional PROMs analysis of subgroups can be found in supplementary file 1 (operatively and non-operatively treated patients) and in supplementary file 2 (Type A, B and C injuries).

**Table 2:** PROMs scores (first column) with a level of recovery at every time point of follow-up compared to pre-injury scores expressed in median (IQR) percentage (second column) and the actual number of patients that has fully recovered (last column)

		PROMs scores* median (IQR)	Level of recovery **(median, IQR)	Number of patients fully recovered *N* (%)
SMFA				
Function index	Pre-injury	95.6 (82.9–98.6)	–	–
	3 months	69.5 (52.6–85.8)	75 (62–92)	42 (45)
	6 months	76.5 (63.6–90.9)	87 (70–99)	49 (59)
	1 year	78.3 (62.5–92.8)	89 (76–99)	48 (61)
	2 years	87.5 (74.3–96.3)	94 (82–99)	33 (75)
Bother index	Pre-injury	95.8 (84.9–100)	–	–
	3 months	66.7 (45.8–85.4)	71 (56–92)	36 (38)
	6 months	75.0 (55.7–91.7)	87 (65–98)	47 (57)
	1 year	79.2 (57.3–92.2)	87 (69–100)	45 (57)
	2 years	87.5 (75.0–97.9)	92 (78–98)	30 (68)
Lower extremity	Pre-injury	98.9 (89.1–100)	–	–
	3 months	70.8 (50.5–91.7)	75 (58–96)	97 (100)
	6 months	79.2 (63.0–93.8)	88 (71–100)	64 (97)
	1 year	81.3 (64.6–95.8)	91 (73–100)	57 (97)
	2 years	87.5 (72.9–97.9)	96 (83–100)	31 (94)
Activities of	Pre-injury	97.5 (80.9–100)	–	–
Daily Living	3 months	57.5 (37.5–79.7)	65 (47–88)	28 (30)
(ADL)	6 months	70.0 (51.6–90.6)	81 (59–97)	37 (45)
	1 year	73.1 (52.8–90.6)	84 (66–99)	39 (49)
	2 years	85.0 (76.5–97.5)	101 (100–106)	39 (89)
Emotion	Pre-injury	90.6 (78.1–96.9)	–	–
	3 months	75.0 (59.4–87.5)	88 (74–97)	54 (57)
	6 months	78.1 (62.5–90.6)	91 (76–100)	56 (68)
	1 year	78.1 (64.8–90.6)	93 (78–100)	51 (65)
	2 years	84.4 (68.8–93.8)	93 (83–100)	30 (68)
EQ-5D	Pre-injury	1.00 (0.85–1.00)	–	–
	6 weeks	0.61 (0.42–0.79)	71 (47–88)	29 (29)
	3 months	0.74 (0.56–0.84)	81 (64–93)	38 (40)
	6 months	0.78 (0.65–0.87)	85 (72–99)	38 (45)
	1 year	0.81 (0.72–0.89)	85 (76–100)	42 (52)
	2 years	0.85 (0.76–1.00)	92 (85–100)	32 (71)

^*^ Median (IQR) scores of the SMFA and EQ-5D

^**^Individual PROMs scores at every time point of follow-up were expressed as a percentage of the pre-injury score. Median percentages (IQR) are presented here

### Factors associated with no full recovery 1 year after the injury

Binary logistic regression analyses revealed that the female gender and a high-energy trauma were independent predictors for not being fully recovered in terms of physical functioning after 1 year of follow-up (Tables 3 and 4). The odds of not recovering on the function index were about three times higher in women compared to men and at least four times higher in patients sustaining a high-energy trauma. The odds of not recovering on the bother index was almost four times higher in females. Female gender and high-energy trauma were also significant predictors for decreased QoL after 1 year (Table 5). The odds of not recovering were about four times higher in female patients and patients with a high-energy trauma. Polytrauma as measured by the ISS (< 16/ ≥ 16) and injury type (AO type A/B/C) did not turn out to be independent predictors for decreased physical functioning and QoL 1 year after the injury.

**Table 3:** Independent predictors for no full recovery of the SMFA function Index at 1 year

	B	OR	95% CI		*P*-value*
Female gender	1.14	3.13	1.12	8.79	**0.03**
High-energy trauma	1.42	4.15	1.29	13.39	**0.017**

Bold values are statistically signficant (*P* < 0.05)

*B* regression coefficient; *OR* Odds ratio; *95% CI* 95% confidence interval

^*^Results on the final model with the *P*-value set at 0.20

**Table 4:** Independent predictors for no full recovery of the SMFA bother Index at 1 year

	B	OR	95% CI		*P*-value*
Age < 65 years	− 0.95	0.39	0.14	1.07	0.06
Female gender	1.27	3.56	1.31	9.70	**0.013**
Complications < 30 days	0.86	2.36	0.78	7.12	0.13

Bold value is statistically signficant (*P* < 0.05)

*B* regression coefficient; *OR* Odds ratio; *95% CI* 95% confidence interval

^*^Results on the final model with the P-value set at 0.20

**Table 5:** Independent predictors for no full recovery of the EQ-5D at 1 year

	*B*	OR	95% CI		*P*-value*
Female gender	1.29	3.64	1.28	10.33	**0.015**
High-energy trauma	1.28	3.60	1.19	10.84	**0.02**
Complications < 30 days	0.73	2.07	0.70	6.09	0.18

Bold values are statistically signficant (*P* < 0.05)

*B* regression coefficient; *OR* Odds ratio; *95% CI* 95% confidence interval

^*^Results on the final model with the P-value set at 0.20

### Patients’ perception of most experienced difficulties during rehabilitation

The individual items of the SMFA to which the patients responded with experiencing severe difficulties (SMFA item score of four or five) were assessed in detail. Subsequently, a top five of encountered difficulties from a patient perspective was composed. A substantial number of patients still experienced limitations in physical activities as well as effects of the injury on their mental wellbeing after, respectively, 3 months and 1 year of follow-up (Table 6). More than half of patients reported severe problems with recreational activities as well as heavy housework or yard work. The latter was still present after 1 year in 31% of patients. Forty-four percent of patients felt physically disabled after 3 months, which gradually decreased to 27% of patients after 1 year.

**Table 6:** Patients’ perception of most experienced difficulties at, respectively, 3 months, 1 year, and 2 years of follow-up after a pelvic ring injury

	SMFA					
	3 months	%*	1 year	%*	2 years	%*
**1**	Recreational activities	54	Heavy house or yard work	31	The effect of doing too much on 1 day	25
**2**	Heavy house work or yard work	53	Bothered by problems with activities around the house	28	Problems performing daily work	21
**3**	Problems performing daily work	44	Problems with bending or kneeling down	28	Heavy house or yard work	18
**4**	Feeling physically disabled	44	Feeling physically disabled	27	Feeling physically disabled	18
**5**	Bothered by problems with recreational activities	41	The effect of doing too much on 1 day	27	Bothered by problems with recreational activities	18

^*^Percentage of patients that experience severe difficulties (SMFA item score of 4 or 5)

Table 7 shows the percentages of patients with their reported difficulties regarding sexual activities from pre-injury up to 2 years after the injury. Most problems were reported at 3 months after the injury and these gradually improved over time.

**Table 7:** Levels of sexual dysfunction at consecutive time points after sustaining a pelvic ring injury

	Pre-injury (*N* = 150)	3 months (*N* = 97)	6 months (*N* = 86)	1 year (*N* = 81)	2 years (*N* = 44)
No problems (%)	85	40	61	58	68
Some problems (%)	6	20	17	19	14
Moderate problems (%)	2	16	6	7	7
Severe problems (%)	1	7	5	9	9
Unable (%)	5	18	12	7	2

## Discussion

In this prospective longitudinal study, we evaluated patient-reported physical functioning and quality of life up to 2 years after a pelvic ring injury and investigated which patient characteristics were predictive of a decreased physical functioning and QoL at 1 year after the injury. Directly after the injury, physical functioning and QoL decrease strongly but then gradually improve up to 2 years after the injury. However, after 2 years, physical functioning as well as QoL are still decreased with a recovery percentage of 75% for physical functioning and 71% for QoL compared to the pre-injury level. Female gender and high-energy trauma are shown to be independent predictors for not fully recovering at 2 year after the pelvic injury. After 3 months, patients experience difficulties with both the physical and mental effects such as difficulties with heavy house or yard work, as well as with feeling physically disabled, which continue to be present after 1 year.

Patients report an evident decrease in physical functioning 3 months after the injury compared to the recalled pre-injury health status. From that moment on, physical functioning keeps on improving up to 2 years after the injury. Between 6 months and 1 year, the recovery curves flatten slightly, while they rise again between one and 2 years, a finding that is in line with previous literature [17]. Median scores on the SMFA function and bother index were, respectively, 76 and 75 out of 100 at 6 months, 78 and 79 at 1 year and both 88 at 2 years of FU. Hoffman et al. [18] reported slightly lower scores using the SMFA in a retrospective cohort study evaluating outcomes after surgically treated lateral compression pelvic ring injuries in 280 patients at 6, 12 and 24 months. Scores on the function, and bother index were, respectively, 72 and 69 at 6 months and improved slightly to 74 and 70 at 1 year and 78 and 76 at 2 years of FU. After 1 and 2 years of follow-up, respectively, 61% and 75% of the patients in our study fully “recovered” in physical functioning (SMFA function index). There are no other prospective studies on recovery of physical function after pelvic ring injuries that use validated PROMs to compare our results with. Next to the reported physical disabilities following a pelvic ring injury, our study showed that patients are also highly affected by the mental consequences as 68% of patients “recovered” on the SMFA bother index and mental & emotional subscale after 2 years.

The high number of patients still being bothered and experiencing mental and emotional problems at 2 years after the injury, highlights the fact that psychological and social effects should also be taken into account to gain an overall picture of the patient’s health perspective. Until recently, subjective emotional disturbances, pain, social and professional consequences have hardly been considered in patients with pelvic ring injuries, even though these injuries can seriously affect QoL [4, 19, 20]. After the pelvic ring injury, an obvious decrease in QoL develops in the first 6 weeks compared to the recalled pre-injury health status. From that moment on, QoL keeps on improving up to 2 years after the injury. Similar to curves on physical functioning, QoL reached a plateau phase between 6 months and 1 year, but rises quite sharply again between one and 2 years. Median EQ-5D scores in our study after 6 months, 1 year and 2 years were, respectively, 0.78, 0.81 and 0.85. Brouwers et al. [17] support these findings, similarly using the EQ-5D to evaluate QoL. They reported a mean EQ-5D scores of 0.77 at 1 year and 0.80 at 2 years and a flattened curve between 6 months and 1 year. Moreover, we showed that after one and 2 years following the injury, “only” 52% and 71% of the patients reported full “recovery” in QoL. Brouwers et al. underline these findings, as they reported that most patients did not achieve their pre-injury state of QoL after 1 year.

Female gender showed to be an independent predictor for achieving no full recovery of physical functioning and QoL 1 year after the injury. However, it is difficult to provide a clear explanation for this finding. Taking a closer look at the demographic characteristics, females were older (mean age 62, SD 23) compared to males (mean age 52, SD 21). They also sustained more type C injuries (15% vs. 9%) and reported higher degrees of sexual dysfunction 1 year after the injury (24% vs. 8%). These findings could be a possible explanation. In other studies, some contradictive results were found regarding the relationship between gender and outcome. One study also reported female gender to be a predictor for decreased QoL [17], whereas another study did not [4]. Polinder et al. and Holbrook et al. [21, 22] found that female gender was a prognostic factor of decreased QoL after general trauma. Independently of injury severity and mechanism, women are reported to show a substantially higher risk of psychological morbidity after major trauma than men with higher rates of post-injury depression, symptoms of acute stress reaction and posttraumatic stress disorder (PTSD) [22]. Female gender was also shown to be a predictor for decreased physical functioning, but only at long-term follow-up (mean of 7 years after the injury) [23]. However, other studies reported no differences between physical functioning in males and females [18] or even improved physical functioning in females [24]. Next to female gender, we also found a high-energy trauma to be a predictive factor for decreased physical functioning and QoL. Brouwers et al. [17] found that ISS was an independent predictor for QoL, a factor closely related to a high-energy trauma as patients often sustain concomitant injuries when sustaining a high-energy trauma. On the other hand, Holstein et al. [4] did not find ISS to be an independent predictor for decreased QoL.

Based on the SMFA questionnaire for physical functioning, we analyzed the factors to which patients experience the most severe difficulties at 3 months and 1 year after the injury. After 3 months, over 40% of patients reported to experience severe difficulties with recreational activities, heavy housework or yard work and daily work. However, the mental impact of the injury such as feeling physically disabled were even so key disabilities. Although the percentage of patients that experienced severe difficulties decreased after 1 year, 30% of patients still report severe physical, as well as mental disabilities. Results of a previous study from our research group evaluating long-term physical functioning and QoL in a large group of patients sustaining a pelvic ring injury, support these findings [19]. At a mean follow-up of 4.4 ± 2.6 years, feeling physically disabled, feeling tired and the effect of doing too much on 1 day affecting the next day were the top three most experienced problems. Difficulties with sexual activities were mostly present at 3 months after the injury and these gradually improved over time.

Some strengths and limitations of this study need to be addressed. The prospective longitudinal design, including recalled pre-injury physical functioning and QoL, is undoubtedly a strength of the present study. With comparable data collected at six different time points, change over time in individual patients could be observed and recall bias avoided. We also reported a high response rate on the PROMs of 82% of the eligible patients. To the best of our knowledge, this is one of the largest prospective longitudinal follow-up studies evaluating physical functioning and QoL after pelvic ring injuries by using validated questionnaires. By comparing PROMs scores at different time points to the pre-injury scores, insight was given in the course of recovery. As a result, both the clinician and subsequently the patient could be provided with valuable information about whether and when to expect a complete recovery. A limitation of this study might be the heterogeneity of the group in terms of age, fracture types, injury severity and presence of associated injuries. However, our study population is an actual reflection of patients with pelvic ring injuries presenting to a large level 1 trauma center. Future research with an even larger sample size, enabling further subgroup analyses, would be preferable. Moreover, 18% of patients passed away within the study period and could therefore not be included in (some of the) follow-up analysis with PROMs. The reported scores could therefore even be an overestimation of the actual perceived physical functioning and QoL since patients with deteriorated health passed away.

## Conclusion

Pelvic ring injuries have a large impact on patient-perceived physical functioning and quality of life. Although both improve over the 2-year period following the injury, only 75% of patients reported to be fully recovered in terms of physical functioning, 68% in terms of being bothered by the injury and 71% in QoL. Female gender and high-energy trauma are independent predictors for patients not fully recovering after 1 year. Most patients experience some mental effects of the injury after both 3 months and 1 year in addition to physical disabilities. The results of this study can be used as a valuable tool by the clinician to inform patients about their expected recovery in terms of physical functioning and QoL in the rehabilitation phase of 2 years after the injury. A multidisciplinary approach covering both the physical and mental aspects of pelvic ring injuries seems appropriate and deserves further attention in prospective research.

## Supplementary Information

Below is the link to the electronic supplementary material.

Supplementary file1 (PDF 77 KB)

Supplementary file2 (PDF 111 KB)

## References

[1] Pohlemann T, Tosounidis G, Bircher M, Giannoudis P, Culemann U (2007). The German multicentre pelvis registry: a template for an european expert network?. Injury.

[2] Verbeek DO, Ponsen KJ, Fiocco M, Amodio S, Leenen LPH, Goslings JC (2017). Pelvic fractures in the Netherlands: epidemiology, characteristics and risk factors for in-hospital mortality in the older and younger population. Eur J Orthop Surg Traumatol.

[3] Fitzgerald CA, Morse BC, Dente CJ (2014). Pelvic ring fractures: Has mortality improved following the implementation of damage control resuscitation?. Am J Surg.

[4] Holstein JH, Pizanis A, Köhler D, Pohlemann T (2013). What are predictors for patients’ quality of life after pelvic ring fractures?. Clin Orthop Relat Res.

[5] Gerbershagen HJ, Dagtekin O, Isenberg J, Martens N, Ozgür E, Krep H, Sabatowski R, Petzke F (2010). Chronic pain and disability after pelvic and acetabular fractures–assessment with the Mainz Pain Staging System. J Trauma.

[6] Tile, Marvin. Helfet, David L. Kellam, James F. Vrahas M (2015) Fractures of the Pelvis and Acetabulum - Principles and Methods of Management.

[7] Banierink H, Ten Duis K, Wendt K, Heineman E, IJpma F, Reininga I (2020) Patient-reported physical functioning and quality of life after pelvic ring injury: A systematic review of the literature. PLoS One 15:e0233226 10.1371/journal.pone.0233226 PMC7367481 32678840

[8] Landelijke Trauma Registratie (LTR). www.lnaz.nl.

[9] Injury severity score (ISS). https://www.aci.health.nsw.gov.au/get-involved/institute-of-trauma-and-injury-management/Data/injury-scoring/injury_severity_score

[10] AO/OTA AO/OTA classification. https://www2.aofoundation.org/wps/portal/surgery.

[11] Swiontkowski MF, Engelberg R, Martin DP, Agel J (1999). Short musculoskeletal function assessment questionnaire: validity, reliability, and responsiveness. J Bone Joint Surg Am.

[12] Reininga IHF, El Moumni M, Bulstra SK, Olthof MGL, Wendt KW, Stevens M (2012). Cross-cultural adaptation of the Dutch Short musculoskeletal function assessment questionnaire (SMFA-NL): Internal consistency, validity, repeatability and responsiveness. Injury.

[13] de Graaf MW, Reininga IHF, Wendt KW, Heineman E, El Moumni M (2019). The short musculoskeletal function assessment: a study of the reliability, construct validity and responsiveness in patients sustaining trauma. Clin Rehabil.

[14] Versteegh M, Vermeulen K, Evers S, de Wit GA, Prenger R, Stolk E (2016). Dutch tariff for the five-level version of EQ-5D. Value Heal.

[15] EuroQol Euroqol (EQ-5D). euroqol.org.

[16] Hung MC, Lu WS, Chen SS, Hou WH, Hsieh CL, Der WJ (2014). Validation of the EQ-5D in patients with traumatic limb injury. J Occup Rehabil.

[17] Brouwers L, De Jongh MAC, De Munter L, Edwards M, Lansink KWW (2020). Prognostic factors and quality of life after pelvic fractures. The Brabant Injury Outcome Surveillance (BIOS) study. PLoS ONE.

[18] Hoffmann MF, Jones CB, Sietsema DL (2012). Persistent impairment after surgically treated lateral compression pelvic injury. Clin Orthop Relat Res.

[19] Banierink H, Reininga IHF, Heineman E, Wendt KW, ten Duis K, IJpma FFA (2019) Long-term physical functioning and quality of life after pelvic ring injuries. Arch Orthop Trauma Surg 0:0 10.1007/s00402-019-03170-2 PMC6687880 30976900

[20] Adelved A, Tötterman A, Glott T, Søberg HL, Madsen JE, Røise O (2014). Patient-reported health minimum 8 years after operatively treated displaced sacral fractures: a prospective cohort study. J Orthop Trauma.

[21] Polinder S, Van Beeck EF, Essink-Bot ML, Toet H, Looman CWN, Mulder S, Meerding WJ (2007). Functional outcome at 2.5, 5, 9, and 24 months after injury in the Netherlands. J Trauma - Inj Infect Crit Care.

[22] Holbrook TL, Hoyt DB (2004). The impact of major trauma: quality-of-life outcomes are worse in women than in men, independent of mechanism and injury severity. J Trauma - Inj Infect Crit Care.

[23] Kokubo Y, Oki H, Sugita D, Takeno K, Miyazaki T, Negoro K, Nakajima H (2017). Functional outcome of patients with unstable pelvic ring fracture. J Orthop Surg.

[24] Ghosh S, Aggarwal S, Kumar P, Kumar V (2019). Functional outcomes in pelvic fractures and the factors affecting them—a short term, prospective observational study at a tertiary care hospital. J Clin Orthop..

