# Supplementary material for: The effects of pelvic ring injuries on quality of life, physical, and mental health: results of a 2-year prospective cohort study
Source: Eur J Trauma Emerg Surg. 2022 Mar 5;48(4):2849–57. doi: 10.1007/s00068-022-01893-3 (PMC9360067; doi:10.1007/s00068-022-01893-3)
Supplement: Supplementary file 1 — Supplementary file1 (PDF 77 KB) [file 68_2022_1893_MOESM1_ESM.pdf]

Supplementary file 1. SMFA and EQ-5D scores of respectively non-operatively and operatively treated patients.

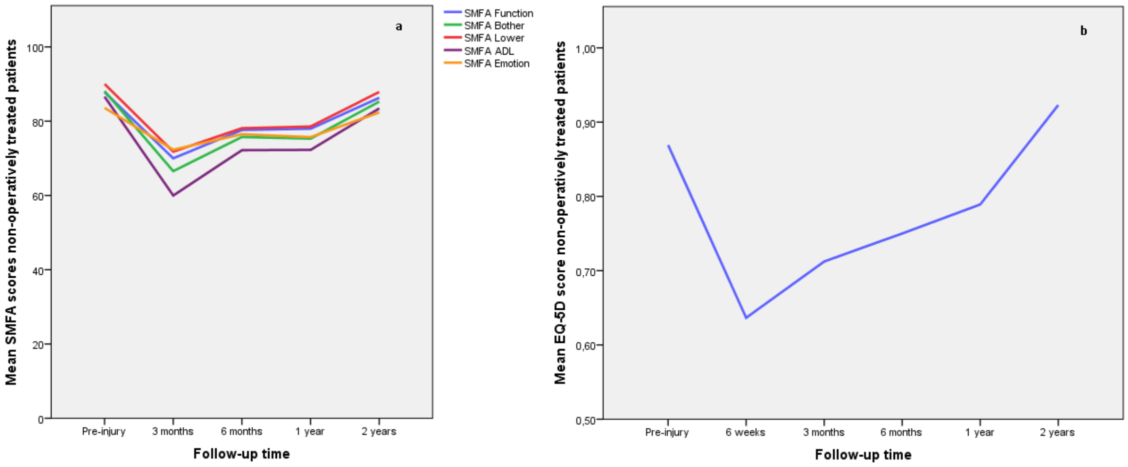

Figure 1. Scores representing recovery of physical functioning (SMFA) and quality of life (EQ-5D) over time after non-operative treatment of pelvic ring injuries (N=244); a) SMFA scores and b) EQ-5D scores. Both the scores are lowest shortly after the injury has occurred at six weeks (EQ-5D) and three months (SMFA) and these gradually improved up to almost the pre-injury level of physical functioning or quality of life at two years after the injury.

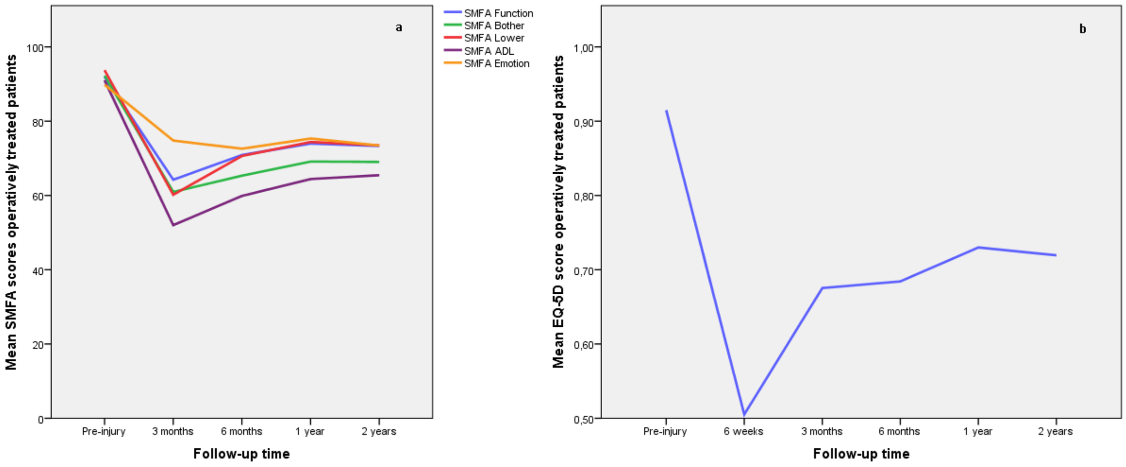

Figure 2. Scores over time after operative treatment of pelvic ring injuries; a) SMFA scores and b) EQ-5D scores. Compared to the non-operatively treated patients (N=244) (figure 1), operatively treated patients report a faster decline in score shortly after the injury at six weeks (EQ-5D) and three months (SMFA). Moreover, the recovery of physical functioning (SMFA) and quality of life (EQ-5D) after operative treatment progresses more gradually compared to the non-operative group, which is probably inherent to the severity of the injury.
