# Supplementary material for: The effects of pelvic ring injuries on quality of life, physical, and mental health: results of a 2-year prospective cohort study
Source: Eur J Trauma Emerg Surg. 2022 Mar 5;48(4):2849–57. doi: 10.1007/s00068-022-01893-3 (PMC9360067; doi:10.1007/s00068-022-01893-3)
Supplement: Supplementary file 2 — Supplementary file2 (PDF 111 KB) [file 68_2022_1893_MOESM2_ESM.pdf]

Supplementary file 2. Outcome of PROMs after respectively type A, type B or type C pelvic ring injuries

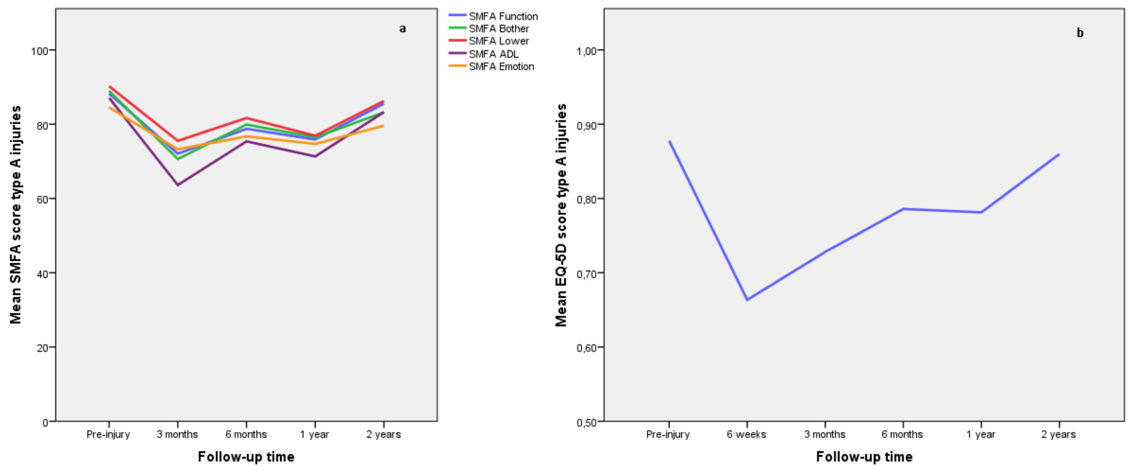

Figure 1. Scores representing recovery of physical functioning (SMFA) and quality of life (EQ-5D) over time of patients with type A pelvic ring injuries (N=117); a) SMFA scores and b) EQ-5D scores. A decrease is seen shortly after the injury at six weeks (EQ-5D) and three months (SMFA and these gradually increased up to two years after the injury.

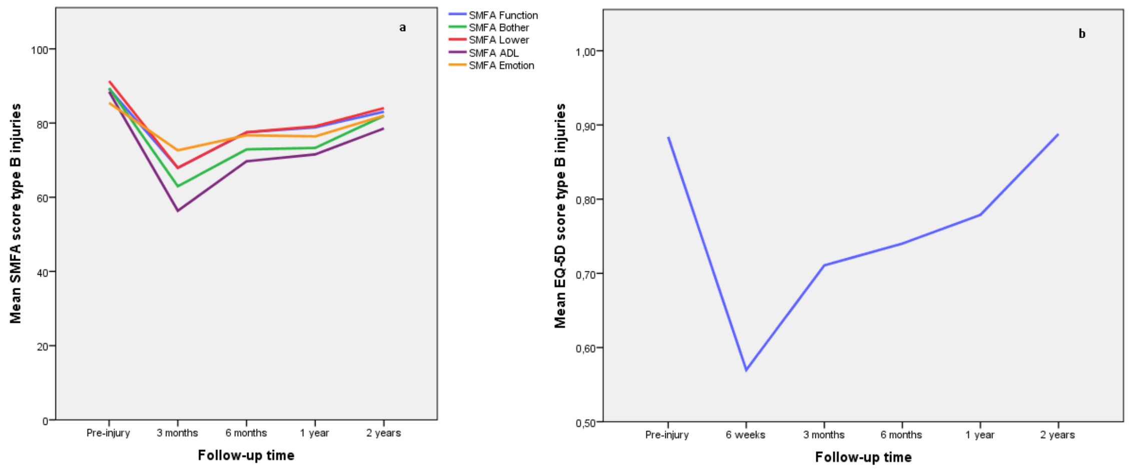

Figure 2. Scores over time of patients with type B pelvic ring injuries (N=144); a) SMFA scores and b) EQ-5D scores. After a decrease shortly after the injury, both scores continued to increase up to two years after the injury.

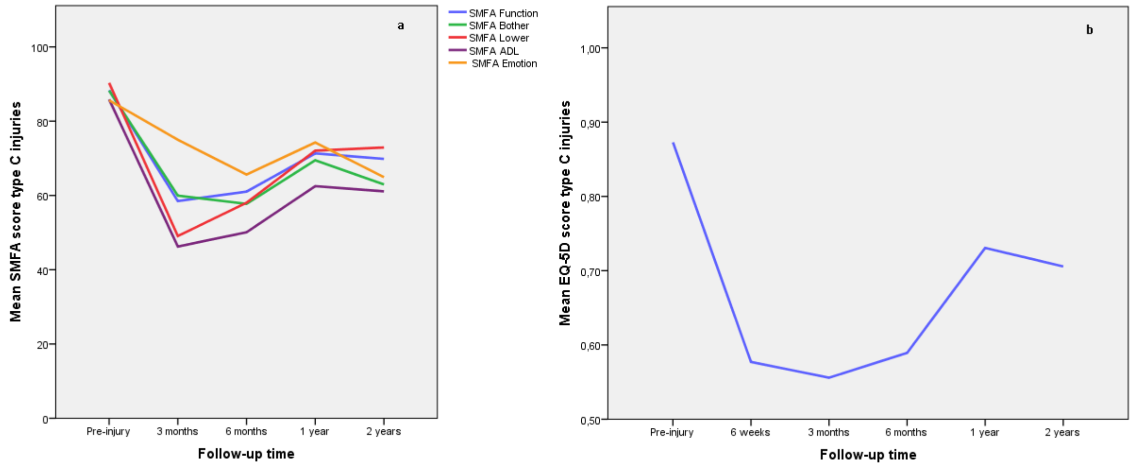

Figure 3. Scores over time of patients with type C pelvic ring injuries (N=36); a) SMFA scores and b) EQ-5D scores. The SMFA scores, indicating physical function (function index, lower extremity and ADL), dropped rapidly shortly after the injury and gradually increased after three months and reach a plateau phase after one year.
